# Proteomic Analysis of *Mecistocirrus digitatus* and *Haemonchus contortus* Intestinal Protein Extracts and Subsequent Efficacy Testing in a Vaccine Trial

**DOI:** 10.1371/journal.pntd.0002909

**Published:** 2014-06-05

**Authors:** Alison J. Dicker, Neil F. Inglis, Erin D. T. Manson, Subhra Subhadra, Manikkavasagan Illangopathy, Raman Muthusamy, David P. Knox

**Affiliations:** 1 Moredun Research Institute, Penicuik, Midlothian, United Kingdom; 2 Department of Veterinary Parasitology, Madras Veterinary College, Tamil Nadu Veterinary and Animal Sciences University, Chennai, India; Queensland Institute of Medical Research, Australia

## Abstract

**Background:**

Gastrointestinal nematode infections, such as *Haemonchus contortus* and *Mecistocirrus digitatus*, are ranked in the top twenty diseases affecting small-holder farmers' livestock, yet research into *M. digitatus*, which infects cattle and buffalo in Asia is limited. Intestine-derived native protein vaccines are effective against *Haemonchus*, yet the protective efficacy of intestine-derived *M. digitatus* proteins has yet to be determined.

**Methodology/Principal Findings:**

A simplified protein extraction protocol (A) is described and compared to an established method (B) for protein extraction from *H. contortus*. Proteomic analysis of the *H. contortus* and *M. digitatus* protein extracts identified putative vaccine antigens including aminopeptidases (H11), zinc metallopeptidases, glutamate dehydrogenase, and apical gut membrane polyproteins. A vaccine trial compared the ability of the *M. digitatus* extract and two different *H. contortus* extracts to protect sheep against *H. contortus* challenge. Both *Haemonchus* fractions (A and B) were highly effective, reducing cumulative Faecal Egg Counts (FEC) by 99.19% and 99.89% and total worm burdens by 87.28% and 93.64% respectively, compared to the unvaccinated controls. There was no effect on *H. contortus* worm burdens following vaccination with the *M. digitatus* extract and the 28.2% reduction in cumulative FEC was not statistically significant. However, FEC were consistently lower in the *M. digitatus* extract vaccinates compared to the un-vaccinated controls from 25 days post-infection.

**Conclusions/Significance:**

Similar, antigenically cross-reactive proteins are found in *H. contortus* and *M. digitatus*; this is the first step towards developing a multivalent native vaccine against *Haemonchus* species and *M. digitatus*. The simplified protein extraction method could form the basis for a locally produced vaccine against *H. contortus* and, possibly *M. digitatus*, in regions where effective cold chains for vaccine distribution are limited. The application of such a vaccine in these regions would reduce the need for anthelmintic treatment and the resultant selection for anthelmintic resistant parasites.

## Author Summary

Parasitic worms infecting the intestines of grazing livestock cause economic losses and welfare problems. Infection is predominantly controlled by wormers, the indiscriminate use of which has led to drug-resistance problems in the worms infecting livestock on which many of the world's resource-poor farmers are dependent. New and cheap control methods are needed. Vaccination with protein extracts from the parasite *Haemonchus contortus* reduces the burden of infection and some work has indicated that cross-protection between closely related parasites is possible. Typically, these extracts are made using relatively sophisticated centrifugation and chromatography equipment as well as needing refrigeration capabilities. In this study, the authors show that equally efficacious extracts can be prepared using a very simplified protocol not requiring these specialist facilities. Proteomic analyses demonstrated the close similarity between protein extracts from both *H. contortus* and *Mecistocirrus digitatus* and vaccine trials in sheep showed that the simplified extraction protocol resulted in an equally efficacious vaccine compared to the more complex methods described prior to this work. Antigenic cross-reactivity was demonstrated between extracts from the two species; the *M. digitatus* extract gave a slight reduction in worm egg output when used to vaccinate sheep challenged with *H. contortus*.

## Introduction

Infections with blood-feeding gastrointestinal nematodes, such as *Haemonchus contortus* and *Mecistocirrus digitatus*, cause significant animal welfare and production losses globally [1], [2]. The latter is an important blood-sucking nematode of cattle in Asia and Central America [3]. In Asia and Africa, where resource-poor small holder farming is more common, gastrointestinal nematode infections of livestock are ranked in the top twenty diseases of livestock affecting the farmers ability to maintain food security and contribute to economic growth [4]. Control of these parasites is currently achieved by the regular use of anthelmintics: However, this approach leads to the inevitable development of anthelmintic resistance [5]. In Tamil Nadu, India, a recent survey (Dicker *et al*, unpublished) found evidence of widespread inefficacy of albendazole, levamisole and ivermectin against *H. contortus* in sheep and goats. As such, novel control strategies, such as vaccines, are urgently needed to enable resource-poor small-holder farmers in Tamil Nadu to control parasite infections in their livestock to ensure their food security.

Substantial progress has been made in identifying several antigens from *H. contortus* which, in their native form, stimulate sufficiently high levels of protective immunity (70–95% reductions in faecal egg output) in the ovine host to indicate that vaccination is feasible [6]–[9]. Much previous work by other authors has focused on proteins or protein complexes expressed on the surface of the worm gut which are exposed to the blood meal and, hence, antibody ingested with it. The antigens generally, but not in all cases, show protease activity and the antibody is thought to mediate protective immunity by blocking the activity of enzymes involved in blood meal digestion within the parasite [10]. The recent increase in genomic data for nematodes such as *Caenorhabditis elegans*, *H. contortus*, hookworms and the trematode *Fasciola hepatica* has allowed identification of novel candidate vaccine antigens whilst proteomics analysis has aided in the identification of post-translational modifications which affect protein folding and protein immunogenicity [11]–[13] (http://nematode.net/NN3_frontpage.cgi). Compared to *H. contortus*, very little is known about *M. digitatus*, with only 25 nucleotide sequences, 12 genes and 38 protein entries present on NCBI (http://www.ncbi.nlm.nih.gov/, 31^st^ May 2013) and no information on the potential for vaccination as an alternative to anthelmintics. Proteomics has been used to investigate other potential vaccine candidates such as excretory/secretory products from adult *Ostertagia ostertagi* and *H. contortus* and larval *Teladorsagia circumcincta* [14]–[17]. No proteomic comparison has been made between extracts from different blood-feeding nematodes but this approach should readily identify if potential vaccine candidates are shared by related species.

Given that anaemia and a reduction in weight gain caused by the haematophagous activity of adult stages seem to be the most important pathogenic effects of *M. digitatus* infection in calves and are similar to those observed during infection with *Haemonchus placei* in calves and *H. contortus* in sheep and goats [2], [18], we sought to compare native protein vaccine preparations, enriched for intestinal surface proteins by Concanavalin A lectin affinity binding [19] from *H. contortus* and *M. digitatus* using proteomics, and to evaluate the protective efficacy of the latter against *H. contortus* challenge in sheep as a prelude to vaccine trials in buffalo in India. Cross-protection has been previously shown to occur in trials conducted in sheep which had been immunized with native *Ostertagia* protein fractions but challenged with *H. contortus*; the *Ostertagia* antigens cross-protected efficiently against *Haemonchus* [20], as such cross-protection between *M. digitatus* and *H. contortus* was believed to be likely.

## Materials and Methods

### Ethics Statement

All experimental procedures were approved by the Moredun Research Institute Experiments and Ethics committee (Experiment number E31/12) and were conducted under the legislation of a UK Home Office License (60/3825) in accordance with the Animals (Scientific Procedures) Act of 1986.

### Parasite Material

Adult *M. digitatus* were collected post mortem from abomasa of cattle collected at an abattoir in Salem, India. Adult *Haemonchus contortus* were obtained from a donor lamb following standard methods as described in Smith & Smith [8]. All parasites were stored at −20°C in 1 X PBS until required.

### Preparation of Protein Fractions

Protein extraction from *M. digitatus* was carried out in India with the resulting protein extract transported to Moredun Research Institute (MRI) whilst maintaining a cold chain; *H. contortus* proteins were purified at MRI. Proteins were extracted using a protocol based on the method in [21]. The parasites were washed several times in 1 X Tris buffered saline (TBS) at a ratio of 10 ml per g dried worms and the worm pellet then homogenised on ice using a chilled pestle and mortar followed by a chilled glass hand homogeniser directly in a 1.0% v/v Triton X-100 buffer. The homogenate was then centrifuged at 2500 X G for 20 mins at 4°C. The supernatant was removed and filter sterilised through a 0.45 µM filter before being mixed with ConA lectin-agarose (Vector laboratories) on a rotary mixer at 4°C for 1 hour. The Protein-ConA-agarose complex was allowed to settle under gravity at 4°C and the supernatant removed. This washing procedure was repeated on 3 occasions using a 0.25% v/v Triton X-100 buffer and then bound proteins were eluted by using a buffer containing 200 mM α Methyl-D-mannopyranoside and 200 mM α Methyl-D-glucopyranoside. The resultant protein solutions were subsequently passed through a 0.22 µM filter. The *H. contortus* extract made using this method was named Hc extract A. A second *H. contortus* extract was made following the method in [20], [19], and is referred to as Hc extract B. Briefly, with centrifugation between each step, adult parasites were extracted in PBS to remove water soluble proteins, then the resultant pellet extracted in PBS/Tween 20 to solubilise membrane-associated proteins with the final pellet solubilised with PBS containing 1.0%v/v Triton X-100. The solution was pumped through a column containing ConA lectin-agarose (Vector laboratories). After thorough washing in a 0.25% v/v Triton X-100 buffer, the column bound proteins were eluted using a buffer containing 200 mM α Methyl-D-mannopyranoside and 200 mM α Methyl-D-glucopyranoside.

Protein concentration was estimated using a Pierce BCA Protein Assay Kit (Thermo Scientific), according to the manufacturer's instructions. An aliquot of each of the *M. digitatus* and *H. contortus* protein elutions were concentrated using the Amicon Ultracel centrifugal filters (Millipore) with a 10 KDa cut off, before a final estimation of protein concentration was obtained.

### Proteomic Analysis of Protein Fractions

To determine the complete protein profile from each parasite, individual non-reduced Novex NuPAGE 4–12% Bis-Tris gels (Life Technologies) for *M. digitatus* and *H. contortus* in 1 X MOPS buffer (Invitrogen) were run at 200 V for 45 mins following the manufacturers' protocols. 2.19 µg and 3.29 µg *M. digitatus* protein and 2.11 µg and 3.17 µg Hc extract A were loaded in NuPAGE LDS sample buffer with the PageRuler Unstained Broad Range Protein Ladder (Fermentas) loaded alongside to allow estimation of protein band size. The gel was stained with SimplyBlue Safestain (Invitrogen) and de-stained with distilled water according to the manufacturer's instructions.

Liquid chromatography-electrospray ionisation-tandem mass spectrometry (LC-ESI-MS/MS) was carried out on the proteins contained in one complete lane from each species at the MRI Proteomics facility using the method as described previously in Wheelhouse *et al* [22] to provide an estimate of the relative abundance of each protein. Mascot generic files were generated and submitted to a local database server, utilising ProteinScape version 2.1 (Bruker Daltonics), to perform database searches against the NCBI non-redundant eukaryotic database (http://www.ncbi.nlm.nih.gov/) and the NEMBASE4 nucleotide database (http://www.nematodes.org/nembase4/index.shtml), using the MASCOT (Matrix science) search algorithm. The carbamidomethyl (C) modification was fixed whilst the Deamidated (NQ) and Oxidation (M) modifications were variable, peptide and fragmentation mass tolerance values were set at 0.5 Da, allowing for a single ^13^C isotope.

Peptide matches were compiled into a protein list compilation (PLC) search result and the quality of proteins inspected manually. Proteins with three or more peptides, or two peptides and with a Molecular Weight Search (MOWSE) score greater than or equal to 90, were deemed significant if at least two different peptides were observed to contain an unbroken run of 4 ‘b’ or ‘y’ ions. The NCBI or NEMBASE protein hit identity for all selected proteins (those passing the quality checks) was determined and the number of identical proteins in each of the databases for both *H. contortus* and *M. digitatus* determined. Proteins identified as mammalian, trypsin or keratin were removed from the analysis.

Subsequently, to determine the identity of individual bands of interest, visible bands were excised from a second gel which had been loaded with 3.29 µg *M. digitatus* extract and 3.17 µg Hc extract A in NuPAGE LDS sample buffer and run as described above to provide identification of individual bands. These individual bands were subjected to LC-ESI-MS/MS and MASCOT searches against both the NCBI non-redundant eukaryotic database and the NEMBASE4 nucleotide database carried out, as described previously. The quality of the protein matches was manually inspected as described for the PLC results.

### Vaccine Trial

A vaccine trial comparing the efficacy of the Hc extract A and *M. digitatus* protein extract against Hc extract B and an unvaccinated control group was undertaken, following standard methods as described in Smith and Smith [8]. Briefly, groups (n = 7) of indoor housed, parasite free lambs, matched for sex and weight, were vaccinated sub-cutaneously three times, three weeks apart with a dose of 40 µg/mL of protein extract (either Hc extract A, Hc extract B or *M. digitatus* extract) in TBS with VAX Saponin adjuvant (Guinness Chemical Products Ltd) at a final concentration of 1 mg/mL. The unvaccinated control group received VAX Saponin adjuvant in TBS only. On the third vaccination day all lambs were challenged with 5000 *H. contortus* L_3_s suspended in water *per os*. From fourteen days post challenge, twice weekly faecal egg counts (FECs) using a modified technique as described in Jackson [23], were carried out on faecal samples obtained *per rectum*. Individual cumulative FEC were estimated by utilising the area under the curve calculation with the linear trapezoidal rule. The mean cumulative FEC for each group was subsequently calculated. Sheep were euthanized on day 35 post challenge, when it was anticipated that all worms present had reached patency, and worms recovered following methods described in Patterson *et al* [24]. Mean total, male and female worm burdens were calculated for each group.

Statistical analysis of the FEC and worm burden results was carried out following the guidelines set out in Coles *et al* [25], data was analysed using Minitab (version 15). The non-parametric Kruskal-Wallis test, followed by Pairwise Mann-Whitney tests, with adjusted P values for multiple comparisons (Bonferroni correction), was used to determine whether statistically significant differences in worm burdens and cumulative FEC were present between the vaccinated groups compared to the unvaccinated control group. S.E.M., range and percentage efficacy (P.E.) for the group mean worm burdens and group mean cumulative FEC were calculated; the P.E. for each vaccinated group was calculated relative to the unvaccinated controls.

### Western Blot of Protein Fractions

2.85 µg *M. digitatus* extract and 2.83 µg Hc extract A in NuPAGE LDS sample buffer were heated at 70°C for 10 mins, then loaded onto a 4–12% Bis-Tris gel (Life Technologies) in 1 X MES buffer (Invitrogen) and run at 200 V for 50 mins following the manufacturers' protocols. 8 µL PageRuler Prestained Protein Ladder (Fermentas) was run alongside the samples, before being removed with a scalpel. The gel was transferred onto a nitrocellulose membrane (Invitrogen) for 1 hour using the XCell blot module, washed twice in a 50 mM Tris, 2.5M NaCl, 0.25% Tween20 pH 7.4 buffer (TNT) before being blocked overnight in TNT. 200 µL sera from each Md extract vaccinated lamb taken 7 days after the third vaccination was pooled together then diluted 1 in 200 in TNT. The blot was incubated in the diluted sera for 1 hour then washed for 10 mins three times in TNT. Monoclonal mouse anti goat/sheep IgG-HRP1 (Sigma-Aldrich) was diluted 1 in 1000 and the blot incubated in it for 1 hour, followed by three 10 mins washes in TNT. Finally the blot was visualised by incubation in DAB reagent (Sigma-Aldrich) until bands became visible.

## Results

### Proteomic Analysis of Protein Fractions

**Figure 1:**
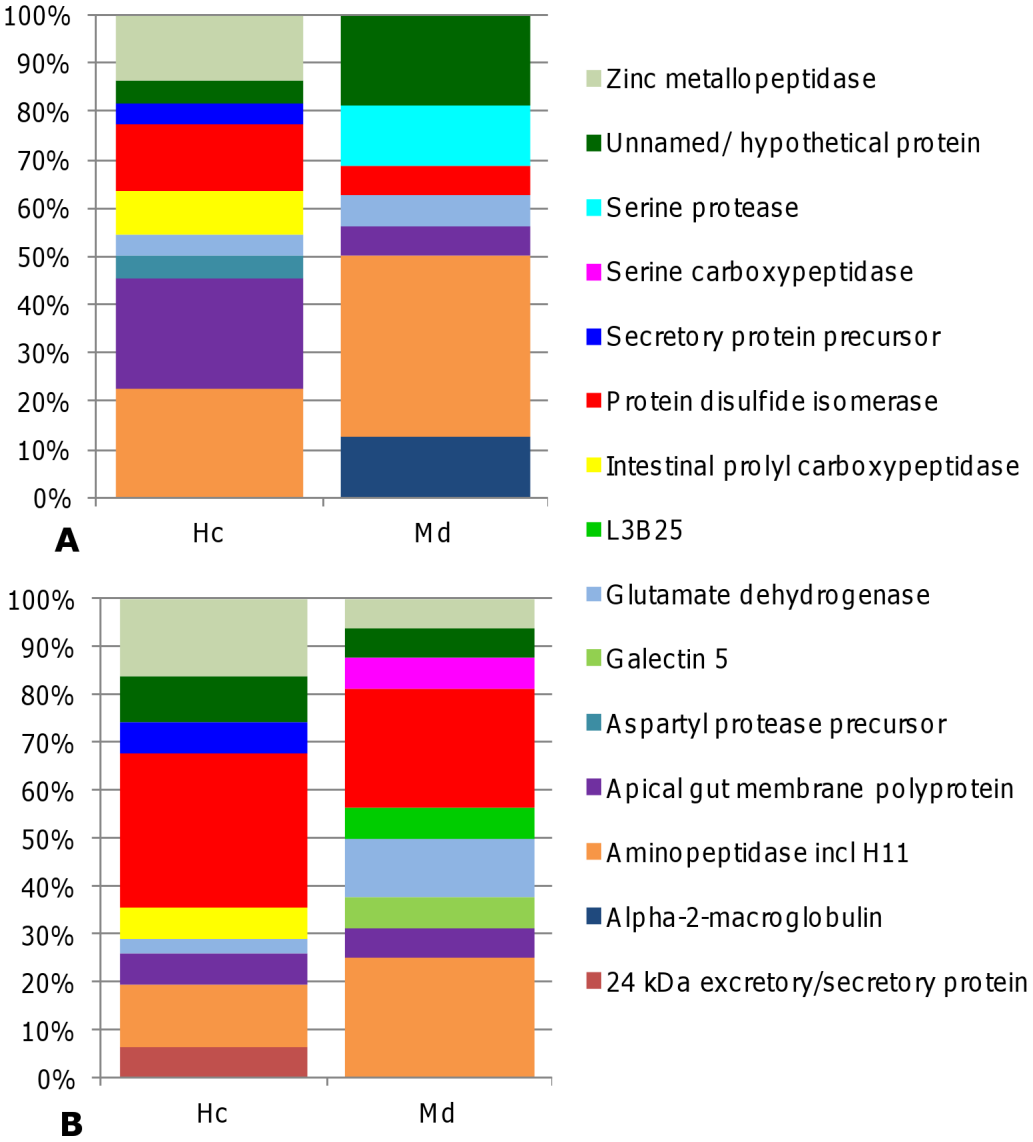
Percentage identity of the selected proteins from each dataset, grouped into similar protein classifications. **A**: NCBI non-redundant eukaryotic database (http://www.ncbi.nlm.nih.gov/) and **B**: NEMBASE4 nucleotide. Each protein identity is expressed as a percentage of the total selected proteins for that dataset.

**Figure 2:**
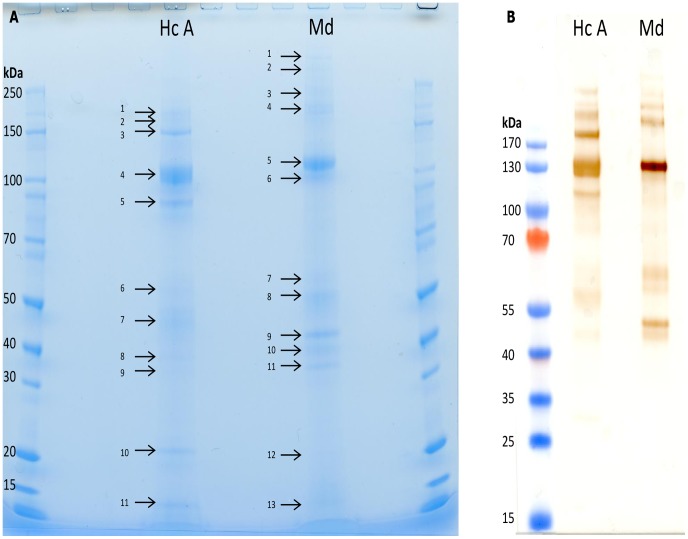
1-D SDS-PAGE gel and western blot of *H. contortus* and *M. digitatus* protein extracts. **A**: 1-D SDS-PAGE gel of 3.17 µg of *H. contortus* protein extract A and 3.29 µg of *M. digitatus* protein extract. Gel was run at 200 V for 45 mins then stained with SimplyBlue® Safestain (Invitrogen). The bands excised from the gel are numbered sequentially and correspond to the bands listed in Tables 1 and 2. **B**: 2.83 µg of *H. contortus* protein extract A and 2.86 µg of *M. digitatus* protein extract were run on a SDS-PAGE gel, transferred onto a nitrocellulose membrane, probed with pooled sera from the *M. digitatus* protein extract vaccinated lambs taken 7 days after vaccination 3 and visualised with monoclonal mouse anti goat/sheep IgG-HRP and DAB.

The relative abundances of different proteins are shown in Figure 1, whilst the identities of individual protein detected in *H. contortus* and *M. digitatus* (Figure 2) are shown in Tables 1 and 2 respectively. The most frequently identified hits in the *H. contortus* NCBInr database (representing 22.7% of the results each) were for aminopeptidase, such as H11 and apical gut membrane polyproteins including the P100GA and P46GA2 proteins (Figure 1). Aminopeptidases were also the most prevalent hit in the *M. digitatus* NCBInr database (37.5%), and the third most prevalent search result (12.9%) in the *H. contortus* NEMBASE nucleotide database. Protein disulphide isomerases and aminopeptidases was the most frequently identified hit (25% each) in the *M. digitatus* NEMBASE nucleotide database. The most frequently identified hit (32.3%) in the *H. contortus* NEMBASE nucleotide database was protein disulphide isomerase, which also accounted for 13.6% of the protein identities from the *H. contortus* NCBInr database search. Zinc metallopeptidases were identified more often from the *H. contortus* database searches, representing 13.64% and 16.1% of the significant hits from the NCBInr and NEMBASE nucleotide searches, respectively, compared to the *M. digitatus* database searches (0% and 6.3%, respectively). In both the *H. contortus* and *M. digitatus* database search results, homologues of other potential vaccine candidates were identified frequently (Figure 1) and included glutamate dehydrogenase [26] and the P100GA proteins [27]. Potential vaccines candidates only identified from *H. contortus* include a 24 kDa excretory/secretory protein [28], aspartyl protease precursor [9] and P46GA2 [27]. Only one potential vaccine component was solely identified from the *M. digitatus* whole lane analysis; a galectin protein 5 identified from the NEMBASE4 nucleotide database search. Between 16 and 41 peptides identified as cysteine proteases (including cathepsins) were present in database searches from both *M. digitatus* and *H. contortus*; however none of the proteins passed the quality checks.

**Table 1:** Putative identities of individual bands from the *H. contortus* protein extract.

Band	Approx Band Size	NCBI Putative Identities	NEMBASE Putative Identities
Hc1	185 kDa	Putative zinc metallopeptidase; *Haemonchus contortus*	Putative zinc metallopeptidase precursor; *Haemonchus contortus*
		Zinc metallopeptidase; *Haemonchus contortus*	Putative zinc metallopeptidase; *Haemonchus contortus*
Hc2	170 kDa	Putative zinc metallopeptidase; *Haemonchus contortus*	Putative zinc metallopeptidase precursor; *Haemonchus contortus*
		Putative zinc metallopeptidase precursor; *Haemonchus contortus*	Putative zinc metallopeptidase; *Haemonchus contortus*
Hc3	145 kDa	No significant hits	no annotation
Hc4	105 kDa	Microsomal aminopeptidase; *Haemonchus contortus*	Microsomal aminopeptidase; *Haemonchus contortus*
		Antigen H11; *Haemonchus contortus*	Membrane aminopeptidase H11-4; *Haemonchus contortus*
		Membrane aminopeptidase H11-4, isoform 4; *Haemonchus contortus*	Aminopeptidase N; *Haemonchus contortus*
Hc5	90 kDa	P46GA2; *Haemonchus contortus*	Apical gut membrane polyprotein; *Haemonchus contortus*
		Apical gut membrane polyprotein; *Haemonchus contortus*	P1a6 protein; *Haemonchus contortus*
		Putative zinc metallopeptidase; *Haemonchus contortus*	Putative zinc metallopeptidase; *Haemonchus contortus*
Hc6	58 kDa	Intestinal prolyl carboxypeptidase; *Haemonchus contortus*	Intestinal prolyl carboxypeptidase; *Haemonchus contortus*
		Disulphide isomerase; *Ostertagia ostertagi*	Protein disulphide isomerase; *Teladorsagia circumcincta*
		Glutamate dehydrogenase; *Haemonchus contortus*	Putative uncharacterized protein; *Dictyocaulus viviparus*
		Calreticulin family protein, partial; *Wuchereria bancrofti*	CBR-CNX-1 protein; *Caenorhabditis briggsae*
			Putative glutamate dehydrogenase; *Haemonchus contortus*
Hc7	47 kDa	Apical gut membrane polyprotein; *Haemonchus contortus*	Apical gut membrane polyprotein; *Haemonchus contortus*
		P100-GA; *Haemonchus contortus*	Putative secretory protein precursor; *Haemonchus contortus*
		Enolase; *Haemonchus contortus*	CE03684 WBGene00011884 locus: enol-1 enolase
Hc8	40 kDa	Protein disulfide isomerase; *Teladorsagia circumcincta*	Protein disulphide isomerase; *Ancylostoma caninum*
		CRE-PDI-2 protein; *Caenorhabditis remanei*	Cathepsin D-like aspartic protease; *Ancylostoma ceylanicum*
		Aspartyl protease precursor; *Haemonchus contortus*	CE03912 WBGene00003053 locus: lmp-1
			Aspartyl protease precursor; *Haemonchus contortus*
Hc9	35 kDa	Galectin 5; *Angiostrongylus cantonensis*	Galectin protein 5; *Caenorhabditis elegans*
			CE29634 WBGene00002268 locus: lec-5
Hc10	22 kDa	24 kDa excretory/secretory protein; *Haemonchus contortus*	24 kDa excretory/secretory protein; *Haemonchus contortus*
		P24; *Haemonchus contortus*	Venom-allergen-like protein 1, isoform a; *Caenorhabditis elegans*
			no annotation
			CE41075 WBGene00022176
Hc11	15 kDa	No significant hits	no annotation
			Putative uncharacterized protein; *Caenorhabditis briggsae*
			CE01221 WBGene00017128

*H. contortus* protein extract run on a 4–12% Bis-Tris gel and bands individually analysed by LC-ESI-MS/MS and MASCOT searches against the NCBInr and NEMBASE4 databases. Only significant hits which were not mammalian, trypsin or keratin are shown, accession numbers for putative hits are shown in Table S1.

**Table 2:** Putative identities of individual bands from the *M. digitatus* protein extract.

Band	Approx Band Size	NCBI Putative Identities	NEMBASE Putative Identities
Md1	>250 kDa	No significant hits	No significant hits
Md2	>250 kDa	No significant hits	No significant hits
Md3	220 kDa	Microsomal aminopeptidase; *Haemonchus contortus*	Microsomal aminopeptidase; *Haemonchus contortus*
			L3B25; *Teladorsagia circumcincta*
Md4	185 kDa	Putative zinc metallopeptidase; *Haemonchus contortus*	Putative zinc metallopeptidase precursor; *Haemonchus contortus*
			L3B25; *Teladorsagia circumcincta*
			Putative uncharacterized protein; *Caenorhabditis briggsae*
			Putative zinc metallopeptidase; *Haemonchus contortus*
Md5	115 kDa	Microsomal aminopeptidase; *Haemonchus contortus*	Microsomal aminopeptidase; *Haemonchus contortus*
		Aminopeptidase N	Aminopeptidase N; *Haemonchus contortus*
		Microsomal aminopeptidase H11; *Haemonchus contortus*	Membrane aminopeptidase H11-4; *Haemonchus contortus*
		Hidden antigen H11; *Haemonchus contortus*	L3B25; *Teladorsagia circumcincta*
		Membrane aminopeptidase H11-4, isoform 4; *Haemonchus contortus*	
Md6	95 kDa	Microsomal aminopeptidase; *Haemonchus contortus*	Microsomal aminopeptidase; *Haemonchus contortus*
			L3B25; *Teladorsagia circumcincta*
Md7	65 kDa	Glutamate dehydrogenase; *Haemonchus contortus*	Putative glutamate dehydrogenase; *Haemonchus contortus*
		Apical gut membrane polyprotein; *Haemonchus contortus*	Apical gut membrane polyprotein; *Haemonchus contortus*
		Protein disulphide isomerase; *Teladorsagia circumcincta*	Putative uncharacterized protein; *Dictyocaulus viviparus*
			L3B25; *Teladorsagia circumcincta*
Md8	60 kDa	Apical gut membrane polyprotein; *Haemonchus contortus*	Apical gut membrane polyprotein; *Haemonchus contortus*
Md9	45 kDa	P100GA2 protein; *Haemonchus contortus*	No significant hits
Md10	40 kDa	Albumin	No significant hits
		Aspartyl protease family member (asp-4); *Caenorhabditis elegans*	
Md11	36 kDa	No significant hits	L3B25; *Teladorsagia circumcincta*
MD12	22 kDa	No significant hits	L3B25; *Teladorsagia circumcincta*
Md13	15 kDa	No significant hits	No significant hits

*M. digitatus* protein extract run on a 4–12% Bis-Tris gel and bands individually analysed by LC-ESI-MS/MS and MASCOT searches against the NCBInr and NEMBASE4 databases. Only significant hits which were not mammalian, trypsin or keratin are shown, accession numbers for putative hits are shown in Table S1.

The proteomic analyses of the individual bands from *H. contortus* and *M. digitatus* protein extracts, excised from a 4–12% Bis Tris gel (Figure 2A) emphasised the similarity between the two parasites, an observation enhanced by the demonstrable antigenic cross-reactivity of the two extracts (Figure 2B). The putative identities for these protein bands are shown in Tables 1 and 2; further details of the proteomic results for these bands can be found in Tables S1 and S2, respectively. Hc1 and 2 and Md4 are all putative zinc metallopeptidases whilst Hc4, Md3, 5 and 6 are all microsomal aminopeptidases or H11: It is possible that Md3, at 220 kDa, is a dimer of either Md5 or 6. Hc7, at approx 47 kDa, and Md9, at approx 45 kDa share a putative protein identity of P100GA whilst Hc8 and Md10 both migrated at approximately 40 kDa and had putative identities of aspartyl proteases. However, other proteins also gave significant matches to these bands indicating that each band may comprise more than one protein, so the identity of these bands could not be confirmed. Hc9 was identified as a Galectin 5, whilst no *M.* digitatus bands were identified as galectins. Combined with the previous, whole lane, analysis this indicates Galectin 5 may be present in both species. Two *H. contortus* bands (Hc3 and 11) and five *M. digitatus* bands (Md1, 2, 11, 12 and 13) returned results either as no significant hits or hypothetical or uncharacterized proteins.

### Vaccine Trial

**Figure 3:**
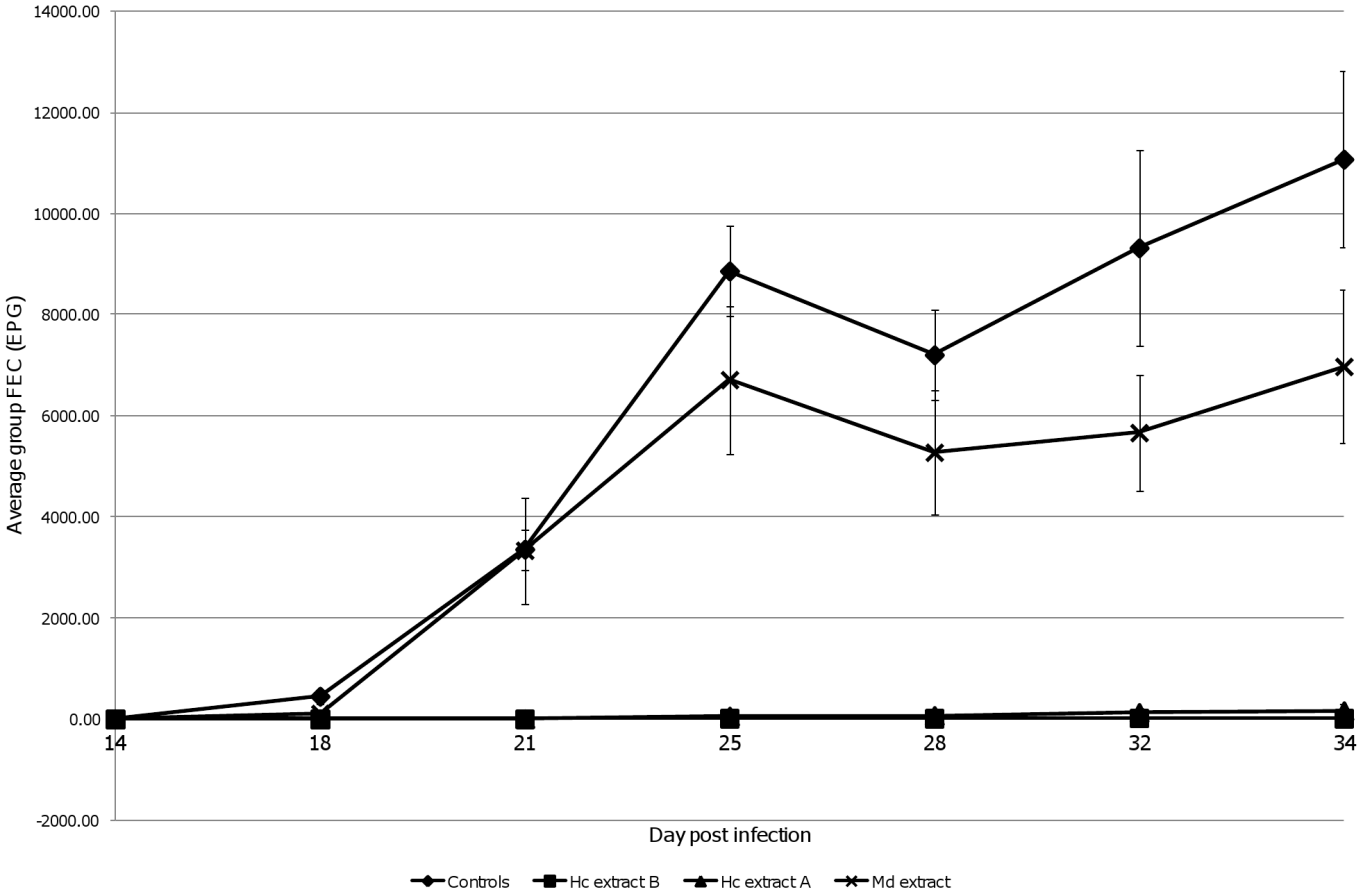
Group mean faecal egg counts (FEC). Average faecal egg count for sheep (group size n = 7) vaccinated with either Hc extract A, Hc extract B, Md extract or unvaccinated control. Error bars show the standard error of the mean (S.E.M.) for each data point.

Figure 3 shows the average FECs for each group obtained from twice weekly *per rectum* faecal samples from day 14 to day 34 post challenge, with the error bars representing the standard error of the mean (S.E.M.) for each group. Both the *H. contortus* vaccine preparations (Hc extracts A and B) elicited similar levels of significant protection against *H. contortus* challenge. The percentage efficacy of the Hc extracts A and B, as determined by group average cumulative FEC, was 99.19%, and 99.89% respectively (Table 3) with the reductions in total worm burdens being 87.28% and 93.64% respectively (Table 4). Both the *H. contortus* vaccine preparations appeared to be more effective against females than males, reducing the worm burden by 94.23% and 79.54% (Hc extract A females and males) and 98.46% and 88.48% (Hc extract B females and males) compared to the unvaccinated controls (Table 4). All cumulative FEC and worm burden reductions for Hc extract A and B vaccinated groups were statistically significant (P<0.0167) compared to the unvaccinated controls, with the P value adjusted for multiple comparisons using Bonferroni correction.

**Table 3:** Mean cumulative faecal egg counts.

Trial group	Mean cumulative FEC	S.E.M. cumulative FEC	Range cumulative FEC	P.E.
Controls	108625	12772	55595–156254	NA
Hc extract B	122***	50	10–334	99.89
Hc extract A	879***	606	24–4442	99.19
Md extract	77992	16235	22583–144992	28.20

Mean cumulative FEC were calculated using the area under the curve, linear trapezoidal method, with standard error of mean (S.E.M.) and range. Percentage efficacy (P.E.) of vaccinated lambs was calculated relative to unvaccinated controls. Results marked with *** are statistically significant compared to unvaccinated controls, (P<0.0167, adjusted for multiple comparisons).

**Table 4:** Mean adult worm burdens.

Trial group	Male		Female		Total	
	Worm count	P.E.	Worm count	P.E.	Worm count	P.E.
Controls	1736 (±187) [1150–2500]	NA	1857 (±193) [1000–2700]	NA	3593 (±332) [2300–5200]	NA
Hc extract B	200*** (±19) [150–250]	88.48	29*** (±15) [0–100]	98.46	229*** (±18) [150–300]	93.64
Hc extract A	350*** (±119) [0–950]	79.54	107*** (±46) [0–350]	94.23	457*** (±147) [0–1100]	87.28
Md extract	2045 (±230) [1500–3200]	−17.83	1586 (±173) [850–2150]	14.62	3631 (±188) [2750–4217]	−1.06

Arithmetic mean (±S.E.M.) [Range] worm counts differentiated into male, female and total worms and percentage efficacy (P.E.) of vaccinated lambs relative to unvaccinated control lambs. Results marked with *** are statistically significant compared to unvaccinated controls, (P<0.0167, adjusted for multiple comparisons).

Figure 3 shows that, although there was an indication that vaccination with *M. digitatus* derived proteins against *H. contortus* challenge reduced the group mean FEC slightly; the cumulative FEC P.E. of 28.20% (Table 3) was not statistically significant. The Md extract vaccinated group average FEC was always lower than the controls; between day 67 and 76 post infection, the FECR was between 24.3% and 39.2% compared to the unvaccinated controls (Figure 3). Average group male and total worm burdens were higher (i.e. negative P.E.) in the Md extract vaccinated group compared to the unvaccinated controls whilst the 14.62% reduction in group mean female worm burden was not statistically significant (Table 4).

## Discussion

The main aim of this work was to compare, using proteomic analyses, the major gut membrane proteins from the closely related haematophagous nematodes, *H. contortus* (affecting sheep and goats) and *M. digitatus* (affecting cattle and buffalo), both of which impose significant constraints on livestock production in tropical and subtropical regions of the world. Gut membrane proteins have proven vaccine efficacy in *H. contortus* [6], and a recent study showed that vaccination of calves with native parasite gut membrane glycoproteins obtained from *H. contortus* conferred protection against both *H. placei* and *H. contortus* [29]. This work, and a previous study [20], indicate that good cross-nematode species protection could be stimulated by vaccination with gut membrane proteins derived from closely related species. Due to their similar haematophagous life cycle, it is possible that cross protection against *M. digitatus* could be achieved utilising a vaccine developed against *H. contortus* [2], [29]. If vaccination is going to be a viable method of control for both *H. contortus* and *M. digitatus* for resource-poor small-holder farmers then the vaccine production method should be cheap without a requirement for specialist and expensive laboratory equipment. This enables local production with minimal dependency on an extensive cold chain. In this paper we describe such a method. Then, the resulting protein extracts from both *M. digitatus* and *H. contortus* were analysed by proteomics to determine whether similar, known candidate vaccine antigens were present in each extract. Finally the protein extracts were tested in a vaccine trial in sheep against *H. contortus* challenge.

Several reports have described the purification of protective antigens from the intestine of *Haemonchus*, and they share the need for successive saline and detergent extractions, high speed centrifugation steps and specialist chromatography equipment [19], [30]–[33]. Redmond *et al* [21] used a simple detergent extract followed by affinity chromatography over Con A lectin to isolate a variety of glycoproteins from *C. elegans*. ConA lectin has a strong affinity for the microvillar surface of the *Haemonchus* intestine [6], [8] and was used in [21] to isolate similar antigens from *C. elegans*, antibody to which cross reacted with numerous *H. contortus* gut antigens. Here, we used the same technique to prepare antigen extracts from *H. contortus* and *M. digitatus* with the modification that the Con A lectin chromatography step was replaced by a simpler protocol of mixing the extract directly with the affinity medium, with an incubation and wash protocol, as described in the materials and methods. The proteomic analyses described underline that this simple method is effective for the isolation of an extract which is highly enriched for intestinal proteins from nematodes and that its composition did not differ obviously from extract B, by comparison to an analysis described by Sherlock [33].

The protein components of each vaccine were determined by both LC-ESI-MS/MS of whole gel lanes (Figure 1) and on individual bands which were excised from the gels (Figure 2A) and then analysed using LC-ESI-MS/MS (Table 1, *Haemonchus* and 2, *Mecistocirrus*). Analysis of the whole gel slices was performed to ensure all proteins present in the extracts would be identified, not just those in sufficient abundance to create a visible band on the gel, whilst the identity of individual bands was determined by excising these from a second gel. There was a broad agreement between these datasets but there tended to be more unidentified or hypothetical protein bands in the analyses from *M. digitatus* compared to *H. contortus*, probably as a result of a lack of specific sequence data for the former. The precise identification of *M. digitatus* proteins is likely to be hampered by low genomic coverage of *M. digitatus* and resultant peptide matches with lower percentage sequence coverage and MOWSE scores [34]. Nonetheless, the results of this analysis indicate that similar proteins were extracted from adult *H. contortus* and *M. digitatus* and, as such, further validation work on the individual proteins identified from *M. digitatus* would be a worthwhile step towards developing a multivalent native vaccine for use in areas where co-infection of livestock with *Haemonchus* species and *M. digitatus* occurs.

Despite some differences in the migration pattern of the protein bands from *M. digitatus* and *H. contortus*, as shown in Figure 2, the analyses here indicate that both extracts are quite similar in terms of protein functions identified. For example, aminopeptidases including H11, zinc metallopeptidases, and protein disulphide isomerases were prominent in both extracts. Many of these proteins have been associated with varying levels of protection against *H. contortus* and other nematodes in vaccine trials [6], [9]. H11 is an insoluble gut membrane glycoprotein of approx 110 kDa involved in blood meal digestion in *H. contortus* and is the most effective vaccine candidate in *H. contortus*, giving greater than 90% reduction in worm burden [30]. The presence of aminopeptidases, including H11, in *M. digitatus* indicates that, as a blood feeder like *H. contortus*, it may also be amenable to the gut antigen vaccination approach. In addition, both extracts contained homologues of zinc metalloproteases, which had been shown previously to be a major component of a host-protective protein complex H-gal-GP [35]. Zinc metallopeptidases were prevalent in the *H. contortus* database searches, representing 13.64% and 16.1% of the significant hits from the NCBInr and NEMBASE nucleotide searches, respectively and were barely detectable in the *M. digitatus* equivalents (0% and 6.3%, respectively). Somewhat surprisingly, there were very few hits to cysteine proteases (none of which passed the proteomic quality checks) despite their apparent abundance in EST datasets from the intestine of *H. contortus* [36], [37]. This anomaly may reflect differences in transcript abundance compared to translation into an actual protein. Gut derived cysteine proteases have been shown to be useful immunogens in *H. contortus* and in human hookworms, which are also blood-feeders [38], [39].

Protein disulphide isomerases were particularly prominent in both datasets; they play important roles in protein folding, catalysing thiol-disulphide interchange which leads to protein disulphide bond formation. In *C. elegans*, they have a role in the formation of cuticular collagen network [14]. Although protein disulphide isomerases have been found in several nematodes and have been detected in ES [14], [15], [40], no report has linked them to the induction of protective immune responses as yet.

Other putative vaccine candidates which have been studied in less detail in parasitic nematodes and which have been identified in this current study include: Glutamate dehydrogenase, the apical gut membrane polyproteins (P100GA and P46GA2), and a 24 kDa excretory/secretory (E/S) protein. Glutamate dehydrogenase has been identified from *H. contortus* and *T circumcincta* using Thiol-Sepharose affinity chromatography but, despite being the major 60 kDa component of the TSBP extract [7], it did not provide protection against infection [31]. Three proteins, P46GA1, P52GA1 and P100GA1 are encoded by the same gene in *H. contortus*, initially being expressed as a polyprotein [27] and together giving 60% and 50% reductions respectively in worm burden and faecal egg outputs in goats [41]. In this analysis, P100GA2 was identified from both *H. contortus* and *M. digitatus* whilst P46GA2 was only identified from *H. contortus*. Finally, a 24 kDa excretory/secretory protein was identified only from the *H. contortus* protein extract. In *H. contortus*, this 24 kDa E/S protein, together with a 15 kDa E/S protein reduced FECs by 32–77% and adult worm burden by 64–85% when tested as a vaccine in sheep [28].

Subsequent to the proteomic analysis, a vaccine trial, comparing the efficacy of the Md extract, Hc extracts A and B against *H. contortus* challenge, was undertaken. Both *H. contortus* vaccine preparations gave statistically significant levels of protection against homologous *H. contortus* challenge, compared to the unvaccinated controls as measured by cumulative FEC and worm burden. The levels of protection (reductions in FEC of 99.19% and 99.89% with Hc extract A and B respectively) exceeding figures of 80% efficacy in 80% of the flock predicted by Barnes *et al* [42] as necessary to provide better protection against infection and disease than standard anthelmintic based control strategies. However, this prediction was made using a computer model based on *Trichostrongylus colubriformis*, a less fecund nematode. These data indicate that the simplified method used to produce Hc extract A could form the basis for a locally produced vaccine against *H. contortus* in regions, such as India, where effective cold chains for vaccine distribution are limited, with the proviso that sufficient worm biomass can be harvested, either from donor animals or abattoir material. All the vaccine preparations tested here were more effective against female worms than their male counterparts, probably reflecting the extra nutritional demands imposed by egg production [43].

*M. digitatus* is not native to the U.K. so performing a protection trial using a crude *M. digitatus* protein extract against homologous challenge was not possible. Therefore, a heterologous challenge with *H. contortus*, which is part of the same *Trichostrongylidae* family and is also haematophagous, was chosen to determine whether protection could be achieved with *M. digitatus* protein extracts [18]. Previously, cross protection trials have been carried out using protein extracts from *Ostertagia ostertagi* and *Teladorsagia circumcincta* against *H. contortus* challenge and using *H. contortus* proteins against *T. circumcincta*, *Trichostrongylus axei* and *Cooperia oncophora* challenge [20], [44]. *O. ostertagi* proteins cross protected against *H. contortus* challenge by reducing FEC by 81–97% and worm burdens by 57–84% [20]. In comparison the results of cross-protection trials against *T. circumcincta*, *Tr. axei* and *C. oncophora* were mixed as *H. contortus* proteins did not provide any protection against infection with any of the aforementioned species, yet *T. circumcincta* proteins caused a significant reduction in FEC (though no effect on worm burdens) following challenge with *H. contortus* [44]. In this current trial, there was no effect on *H. contortus* worm burdens following vaccination with a *M. digitatus* vaccine extract and the 28.2% reduction in cumulative FEC was not statistically significant. However, it is notable that the FEC were consistently lower in the *M. digitatus* extract vaccinates compared to the challenge controls from 25 days post-infection. A trial is now in progress in India to determine whether *M. digitatus* proteins provide protection against *M. digitatus* challenge. The failure to obtain evidence of effective cross-protection was somewhat surprising given the evidence from prior studies discussed above and that the parasites are closely related and obligate blood feeders. Moreover, the immunoblot data shown in Figure 2 confirms that there is strong antigenic cross reactivity between the *H. contortus* and *M. digitatus* extracts. Perhaps the explanation lies in the relative abundance or absence of specific components when the two vaccine extracts are compared. For example, there is solid evidence that the zinc metallopeptidases contribute to vaccine-induced immunity against *Haemonchus* but these were much less abundant in *M. digitatus* (Figure 1) and the 24 kDa *Haemonchus* ES protein, associated with strong protective immune responses [28] was not detected in the *M. digitatus* extract.

## Supporting Information

Table S1 **Putative identities of individual bands from the** ***H. contortus*** **protein extract.** *H. contortus* protein extract run on a 4–12% Bis-Tris gel and bands individually analysed by LC-ESI-MS/MS and MASCOT searches against the NCBInr and NEMBASE4 databases. Only significant hits which were not mammalian, trypsin or keratin are shown. Table showing accession numbers, number of peptide matches, MOWSE scores and sequence identities of each protein match.
(DOCX)

Table S2 **Putative identities of individual bands from the** ***M. digitatus*** **protein extract.** *M. digitatus* protein extract run on a 4–12% Bis-Tris gel and bands individually analysed by LC-ESI-MS/MS and MASCOT searches against the NCBInr and NEMBASE4 databases. Only significant hits which were not mammalian, trypsin or keratin are shown. Table showing accession numbers, number of peptide matches, MOWSE scores and sequence identities of each protein match.
(DOCX)

## References

[1] Molento M (2009) Parasite control in the age of drug resistance and changing agricultural practices. Vet Parasitol 163: 229–234. 19560869 10.1016/j.vetpar.2009.06.007

[2] van Aken D, Vercruysse J, Dargantes AP, Lagapa JT, Raes S, et al (1997) Pathophysiological aspects of *Mecistocirrus digitatus* (Nematoda: trichostrongylidae) infection in calves. Vet Parasitol 69: 255–263. 9195735 10.1016/s0304-4017(96)01132-6

[3] Soulsby EJL (1982) Helminths, arthropods and protozoa of domesticated animals: Bailliere Tindall, London.

[4] Perry BD, Randolph TF, McDermott JJ, Sones KR, Thornton PK (2002) Investing in animal health research to alleviate poverty. ILRI (International Livestock Research Institute), Nairobi, Kenya.

[5] Wolstenholme AJ, Fairweather I, Prichard R, von Samson-Himmelstjerna G, Sangster NC (2004) Drug resistance in veterinary helminths. Trends Parasitol 20: 469–476. 15363440 10.1016/j.pt.2004.07.010

[6] Knox DP, Smith WD (2001) Vaccination against gastrointestinal nematode parasites of ruminants using gut-expressed antigens. Vet Parasitol 100: 21–32. 11522403 10.1016/s0304-4017(01)00480-0

[7] Knox DP, Smith SK, Smith WD (1999) Immunization with an affinity purified protein extract from the adult parasite protects lambs against infection with *Haemonchus contortus* . Parasite Immunol 21: 201–210. 10320617 10.1046/j.1365-3024.1999.00220.x

[8] Smith WD, Smith SK (1993) Evaluation of aspects of the protection afforded to sheep immunised with a gut membrane protein of *Haemonchus contortus* . Res Vet Sci 55: 1–9. 8378601 10.1016/0034-5288(93)90025-b

[9] Knox D (2011) Proteases in blood-feeding nematodes and their potential as vaccine candidates. Adv Exp Med Biol 712: 155–176. 21660664 10.1007/978-1-4419-8414-2_10

[10] Smith WD, Zarlenga DS (2006) Developments and hurdles in generating vaccines for controlling helminth parasites of grazing ruminants. Vet Parasitol 139: 347–359. 16750599 10.1016/j.vetpar.2006.04.024

[11] Barrett J, Jefferies JR, Brophy PM (2000) Parasite Proteomics. Parasitol Today 16: 400–403. 10951601 10.1016/s0169-4758(00)01739-7

[12] Wheeler DL, Barrett T, Benson DA, Bryant SH, Canese K, et al (2008) Database resources of the National Center for Biotechnology Information. Nucleic Acids Res 36: D13–D21. 18045790 10.1093/nar/gkm1000 PMC2238880

[13] Dalton JP, Brindley PJ, Knox DP, Brady CP, Hotez PJ, et al (2003) Helminth vaccines: from mining genomic information for vaccine targets to systems used for protein expression. Int J Parasitol 33: 621–640. 12782060 10.1016/s0020-7519(03)00057-2

[14] Geldhof P, Vercauteren I, Knox D, Demaere V, Van Zeveren AM, et al (2003) Protein disulphide isomerase of *Ostertagia ostertagi*: an excretory- secretory product of L4 and adult worms? Int J Parasitol 33: 129–136. 12633650 10.1016/s0020-7519(02)00260-6

[15] Craig H, Wastling J, Knox DP (2006) A preliminary proteomic survey of the in vitro excretory/secretory products of fourth-stage larval and adult *Teladorsagia circumcincta* . Parasitology 132: 535–543. 16388693 10.1017/S0031182005009510

[16] Smith SK, Nisbet AJ, Meikle LI, Inglis NF, Sales J, et al (2009) Proteomic analysis of excretory/secretory products released by *Teladorsagia circumcincta* larvae early post-infection. Parasite Immunol 31: 10–19. 19121079 10.1111/j.1365-3024.2008.01067.x

[17] Yan F, Xu L, Liu L, Yan R, Song X, et al (2010) Immunoproteomic analysis of whole proteins from male and female adult *Haemonchus contortus* . Vet J 185: 174–179. 19560953 10.1016/j.tvjl.2009.05.021

[18] Taylor MA, Coop RL, Wall RL (2007) Veterinary Parasitology: Blackwell Publishing Ltd.

[19] Smith WD (1993) Protection in lambs immunized with *Haemonchus contortus* gut membrane proteins. Res Vet Sci 54: 94–101. 8434155 10.1016/0034-5288(93)90017-a

[20] Smith WD, Smith SK, Pettit D (2000) Evaluation of immunization with gut membrane glycoproteins of *Ostertagia ostertagi* against homologous challenge in calves and against *Haemonchus contortus* in sheep. Parasite Immunol 22: 239–247. 10792763 10.1046/j.1365-3024.2000.00303.x

[21] Redmond DL, Geldhof P, Knox DP (2004) Evaluation of *Caenorhabditis elegans* glycoproteins as protective immunogens against *Haemonchus contortus* challenge in sheep. Int J Parasitol 34: 1347–1353. 15542095 10.1016/j.ijpara.2004.08.013

[22] Wheelhouse NM, Sait M, Aitchison K, Livingstone M, Wright F, et al (2012) Processing of *Chlamydia abortus* polymorphic membrane protein 18D during the chlamydial developmental cycle. PLoS One 7: e49190. 23145118 10.1371/journal.pone.0049190 PMC3493501

[23] Jackson F (1974) New technique for obtaining nematode ova from sheep faeces. Lab Pract 23: 65–66. 4856197

[24] Patterson DM, Jackson F, Huntley JF, Stevenson LM, Jones DG, et al (1996) Studies on caprine responsiveness to nematodiasis: segregation of male goats into responders and non-responders. Int J Parasitol 26: 187–194. 8690543 10.1016/0020-7519(95)00121-2

[25] Coles GC, Bauer C, Borgsteede FHM, Geerts S, Klei TR, et al (1992) World Association for the Advancement of Veterinary Parasitology (WAAVP) methods for the detection of anthelmintic resistance in nematodes of veterinary importance. Vet Parasitol 44: 35–44. 1441190 10.1016/0304-4017(92)90141-u

[26] Skuce PJ, Stewart EM, Smith WD, Knox DP (1999) Cloning and characterization of glutamate dehydrogenase (GDH) from the gut of *Haemonchus contortus* . Parasitology 118: 297–304. 10205806 10.1017/s0031182098003850

[27] Jasmer DP, Perryman LE, McGuire TC (1996) *Haemonchus contortus* GA1antigens: Related, phospholipase C-sensitive, apical gut membrane proteins encoded as a polyprotein and released from the nematode during infection. Proc Nat Acad Sci U S A 93: 8642–8647. 10.1073/pnas.93.16.8642 PMC38726 8710924

[28] Schallig HDFH, van Leeuwen MAW, Cornelissen AWCA (1997) Protective immunity induced by vaccination with two *Haemonchus contortus* excretory secretory proteins in sheep. Parasite Immunol 19: 447–453. 9372512 10.1046/j.1365-3024.1997.d01-148.x

[29] Bassetto CC, Silva BF, Newlands GFJ, Smith WD, Amarante AFT (2011) Protection of calves againast *Haemonchus placei* and *Haemonchus contortus* after immunization with gut membrane proteins from *H. contortus* . Parasite Immunol 33: 377–381. 21535018 10.1111/j.1365-3024.2011.01295.x

[30] Munn EA, Smith TS, Smith H, Fiona MJ, Smith FC, et al (1997) Vaccination against *Haemonchus contortus* with denatured forms of the protective antigen H11. Parasite Immunol 19: 243–248. 9364553 10.1046/j.1365-3024.1997.d01-205.x

[31] Knox DP, Smith SK, Redmond DL, Smith WD (2005) Protection induced by vaccinating sheep with a thiol-binding extract of *Haemonchus contortus* membranes is associated with its protease components. Parasite Immunol 27: 121–126. 15910420 10.1111/j.1365-3024.2005.00750.x

[32] Smith TS, Munn EA (1990) Strategies for vaccination against gastro-intestinal nematodes. Rev Sci Tech 9: 577–595. 2132697 10.20506/rst.9.2.495

[33] Sherlock PA (2007) Studies on protective integral gut membrane glycoprotein fractions from *Haemonchus contortus*: MSc Thesis, University of Edinburgh. 146 p.

[34] Millares P, LaCourse EJ, Perally S, Ward DA, Prescott MC, et al (2012) Proteomic profiling and protein identification by MALDI-TOF mass spectrometry in unsequenced parasitc nematodes. PLoS One 7: e33590. 22479418 10.1371/journal.pone.0033590 PMC3315570

[35] Newlands GFJ, Skuce PJ, Nisbet AJ, Redmond DL, Smith SK, et al (2006) Molecular characterization of a family of metalloendopeptidases from the intestinal brush border of *Haemonchus contortus* . Parasitology 133: 357–368. 16740178 10.1017/S0031182006000217

[36] Shompole S, Jasmer DP (2001) Cathepsin B-like cysteine proteaes confer intestinal cysteine protease activity in *Haemonchus contortus* . J Biol Chem 276: 2928–2934. 11032834 10.1074/jbc.M007321200

[37] Jasmer DP, Mitreva MD, McCarter JP (2004) mRNA sequences for *Haemonchus contortus* intestinal cathepsin B-like cysteine proteases display an extreme in abundance and diversity compared with other adult mammalian parasitic nematodes. Mol Biochem Parasitol 137: 297–305. 15383300 10.1016/j.molbiopara.2004.06.010

[38] Redmond DL, Knox DP (2004) Protection studies in sheep using affinity-purified and recombinant cysteine proteinases of adult *Haemonchus contortus* . Vaccine 22: 4252–4261. 15474716 10.1016/j.vaccine.2004.04.028

[39] Loukas A, Bethony JM, Williamson AL, Goud GN, Mendez S, et al (2004) Vaccination of dogs with a recombinant cysteine protease from the intestine of canine hookworms diminishes the fecundity and growth of worms. J Infect Dis 189: 1952–1961. 15122534 10.1086/386346

[40] Vercauteren I, Geldhof P, Peelaers I, Claerebout E, Berx G, et al (2003) Identification of excretory-secretory products of larval and adult *Ostertagia ostertagi* by immunoscreening of cDNA libraries. Mol Biochem Parasitol 126: 201–208. 12615319 10.1016/s0166-6851(02)00274-8

[41] Jasmer DP, Perryman LE, Conder GA, Crow S, McGuire TC (1993) Protective immunity to *Haemonchus contortus* induced by immunoaffinity isolated antigens that share a phylogenetically conserved carbohydrate gut surface epitope. J Immunol 151: 5450–5460. 7693812

[42] Barnes EH, Dobson RJ, Barger IA (1995) Worm control and anthelmintic resistance - Adventures with a model. Parasitol Today 11: 56–63. 15275374 10.1016/0169-4758(95)80117-0

[43] Martin CJ, Clunies R (1934) A minimal computation of blood removed daily by *Haemonchus contortus* . J Helminthol 12: 137–142.

[44] Smith WD, Pettit D, Smith SK (2001) Cross-protection studies with gut membrane glycoprotein antigens from *Haemonchus contortus* and *Teladorsagia circumcincta* . Parasite Immunol 23: 203–211. 11298297 10.1046/j.1365-3024.2001.00375.x

