# Supplementary material for: Proteomic Analysis of Mecistocirrus digitatus and Haemonchus contortus Intestinal Protein Extracts and Subsequent Efficacy Testing in a Vaccine Trial
Source: PLoS Negl Trop Dis. 2014 Jun 5;8(6):e2909. doi: 10.1371/journal.pntd.0002909 (PMC4046941; doi:10.1371/journal.pntd.0002909)
Supplement: Table S1 — Putative identities of individual bands from the H. contortus protein extract. H. contortus protein extract run on a 4–12% Bis-Tris gel and bands individually analysed by LC-ESI-MS/MS and MASCOT searches against the NCBInr and NEMBASE4 databases. Only significant hits which were not mammalian, trypsin or keratin are shown. Table showing accession numbers, number of peptide matches, MOWSE scores and sequence identities of each protein match. (DOCX) [file pntd.0002909.s001.docx]

**Table S1. Putative identities of individual bands from the *H. contortus* protein extract.**

| **Band** | **Approx band size** | **NCBI Results** | | | | | | | **NEMBASE Results** | | | | | |
| --- | --- | --- | --- | --- | --- | --- | --- | --- | --- | --- | --- | --- | --- | --- |
|  |  | **Accession number** | | **Putative Identity** | | **Peptides** | **MOWSE score** | **Sequence coverage (%)** | **Accession number** | **Putative Identity** | **Peptides** | **MOWSE score** | **Sequence coverage (%)** | |
| Hc1 | 185kDa | gi\|3415005 | | Putative zinc metallopeptidase; *Haemonchus contortus* | | 13 | 748.6 | 21 | HCC00065_1 | Putative zinc metallopeptidase precursor; *Haemonchus contortus* | 13 | 731.1 | 4.1 | |
|  |  | gi\|1483338 | | Zinc metallopeptidase; *Haemonchus contortus* | | 12 | 690 | 18.5 | HCC00087_1 | Putative zinc metallopeptidase; *Haemonchus contortus* | 10 | 422.2 | 6.1 | |
|  |  | gi\|3388169 | | Putative zinc metallopeptidase; *Haemonchus contortus* | | 7 | 403.6 | 19 | HCC09875_1 | Putative zinc metallopeptidase; *Haemonchus contortus* | 6 | 301 | 6.3 | |
|  | | | | | | | | | HCC09809_1 | Putative zinc metallopeptidase; *Haemonchus contortus* | 4 | 222.1 | 6.1 | |
|  | | | | | | | | | HCC09838_2 | Putative zinc metallopeptidase; *Haemonchus contortus* | 3 | 97.8 | 3.1 | |
|  | | | | | | | | | HCC07548_1 | Putative zinc metallopeptidase; *Haemonchus contortus* | 2 | 97.1 | 4.6 | |
| Hc2 | 170kDa | gi\|3415005 | Putative zinc metallopeptidase; *Haemonchus contortus* | | | 18 | 1007.4 | 25.8 | HCC00065_1 | Putative zinc metallopeptidase precursor; *Haemonchus contortus* | 17 | 852 | 6.6 | |
|  |  | gi\|2905790 | Putative zinc metallopeptidase precursor; *Haemonchus contortus* | | | 12 | 797.2 | 28.2 | HCC00087_1 | Putative zinc metallopeptidase; *Haemonchus contortus* | 8 | 375.4 | 4.1 | |
|  |  | gi\|3388169 | Putative zinc metallopeptidase; *Haemonchus contortus* | | | 8 | 400.2 | 17.1 | HCC09875_1 | Putative zinc metallopeptidase; *Haemonchus contortus* | 8 | 336.2 | 9 | |
|  |  | gi\|5281311 | Putative zinc metallopeptidase; *Haemonchus contortus* | | | 3 | 162.8 | 4.9 | HCC09809_1 | Putative zinc metallopeptidase; *Haemonchus contortus* | 5 | 283.3 | 6.1 | |
|  | | | | | | | | | HCC09838_2 | Putative zinc metallopeptidase; *Haemonchus contortus* | 3 | 199.5 | 6.8 | |
|  | | | | | | | | | HCC00092_1 | Putative zinc metallopeptidase; *Haemonchus contortus* | 3 | 162.8 | 1.1 | |
| Hc3 | 145kDa | No significant hits | | | | | | | HCC02525_1 | no annotation | 6 | 331.4 | 13 | |
|  | | | | | | | | | HCC02737_1 | no annotation | 4 | 167.5 | 11.3 | |
| Hc4 | 105kDa | gi\|6012985 | Microsomal aminopeptidase; *Haemonchus contortus* | | 49 | | 2963.9 | 52.4 | HCC00035_1 | Microsomal aminopeptidase; *Haemonchus contortus* | 51 | 3049.5 | 15.0 | |
|  |  | gi\|6012987 | Microsomal aminopeptidase; *Haemonchus contortus* | | 36 | | 2311.0 | 39.8 | HCC00036_1 | Microsomal aminopeptidase; *Haemonchus contortus* | 39 | 2256.0 | 10.9 | |
|  |  | gi\|55977178 | Antigen H11; *Haemonchus contortus* | | 32 | | 1942.7 | 40.7 | HCC01491_1 | Membrane aminopeptidase H11-4; *Haemonchus contortus* | 34 | 1927.9 | 11.7 | |
|  |  | gi\|14140052 | Membrane aminopeptidase H11-4, isoform 4; *Haemonchus contortus* | | 32 | | 1830.4 | 38.4 | HCC00029_1 | Aminopeptidase N; *Haemonchus contortus* | 31 | 1945.6 | 11.0 | |
|  |  | gi\|2499899 | Membrane glycoprotein H11 | | 31 | | 1922.2 | 41.2 | HCC01156_1 | Membrane aminopeptidase H11-4; *Haemonchus contortus* | 3 | 136.8 | 4.6 | |
|  |  | gi\|29825703 | Hidden antigen H11; *Haemonchus contortus* | | 30 | | 1861.6 | 38.2 |  | | | | | |
|  |  | gi\|218139543 | Microsomal aminopeptidase H11; *Haemonchus contortus* | | 30 | | 1839.6 | 40.2 |  | | | | | |
| Hc5 | 90kDa | gi\|218921786 | P46GA2.1A; *Haemonchus contortus* | | 22 | | 1221.3 | 57.6 | HCC00001_2 | Apical gut membrane polyprotein; *Haemonchus contortus* | 18 | 1012.2 | | 6.5 |
|  |  | gi\|1335976 | Apical gut membrane polyprotein; *Haemonchus contortus* | | 18 | | 969.4 | 21.7 | HCC00191_1 | P1a6 protein; *Haemonchus contortus* | 11 | 753.6 | | 16 |
|  |  | gi\|218921788 | P46GA2.1B; *Haemonchus contortus* | | 17 | | 953.8 | 44.1 | HCC09809_1 | Putative zinc metallopeptidase; *Haemonchus contortus* | 4 | 142.7 | | 6.1 |
|  |  | gi\|3415005 | Putative zinc metallopeptidase; *Haemonchus contortus* | | 5 | | 283.3 | 10.8 | HCC07548_1 | Putative zinc metallopeptidase; *Haemonchus contortus* | 2 | 112.6 | | 4.6 |
|  |  | gi\|5281311 | Putative zinc metallopeptidase; *Haemonchus contortus* | | 2 | | 110.1 | 4.4 | HCC09838_2 | Putative zinc metallopeptidase; *Haemonchus contortus* | 2 | 93.0 | | 3.1 |
| Hc6 | 58kDa | gi\|146425171 | Intestinal prolyl carboxypeptidase 2; *Haemonchus contortus* | | 11 | | 621.4 | 18.5 | HCC00298_2 | Intestinal prolyl carboxypeptidase 2; *Haemonchus contortus* | 9 | 381.4 | | 5.0 |
|  |  | gi\|146425169 | Intestinal prolyl carboxypeptidase 1; *Haemonchus contortus* | | 3 | | 366.5 | 11.8 | HCC00298_1 | Intestinal prolyl carboxypeptidase 2; *Haemonchus contortus* | 8 | 426.9 | | 5.1 |
|  |  | gi\|16945685 | Disulphide isomerase; *Ostertagia ostertagi* | | 6 | | 365.8 | 18.3 | OOC00150_1 | Protein disulphide isomerase; *Teladorsagia circumcincta* | 5 | 268.8 | | 5.8 |
|  |  | gi\|253721983 | Glutamate dehydrogenase; *Haemonchus contortus* | | 3 | | 185.5 | 6.4 | HCC00273_1 | Protein disulphide isomerase; *Teladorsagia circumcincta* | 4 | 282.8 | | 8.5 |
|  |  | gi\|402590577 | Calreticulin family protein, partial; *Wuchereria bancrofti* | | 3 | | 182.8 | 6.4 | DVC00633_1 | Putative uncharacterized protein; *Dictyocaulus viviparus* | 4 | 222.4 | | 3.5 |
|  |  | gi\|15209369 | Disulphide isomerase; *Ostertagia ostertagi* | | 2 | | 168.8 | 18.2 | ACC15035_1 | CBR-CNX-1 protein; *Caenorhabditis briggsae* | 4 | 216.4 | | 2.7 |
|  | | | | | | | | | HCC00232_3 | Intestinal prolyl carboxypeptidase 1; *Haemonchus contortus* | 4 | 205.9 | | 2.6 |
|  | | | | | | | | | ACC00840_1 | Protein disulphide isomerase; *Ancylostoma caninum* | 3 | 213.0 | | 2.6 |
|  | | | | | | | | | HCC00006_2 | Putative glutamate dehydrogenase; *Haemonchus contortus* | 3 | 202.0 | | 2.4 |
|  | | | | | | | | | HCC00318_1 | Intestinal prolyl carboxypeptidase 1; *Haemonchus contortus* | 2 | 197.1 | | 5.2 |
| Hc7 | 47kDa | gi\|1335976 | Apical gut membrane polyprotein; *Haemonchus contortus* | | 17 | | 1117.6 | 33.8 | HCC00001_2 | Apical gut membrane polyprotein; *Haemonchus contortus* | 22 | 1353.6 | | 11.3 |
|  |  | gi\|218921784 | P100-GA; *Haemonchus contortus* | | 16 | | 1048.4 | 34.9 | HCC00272_1 | Putative secretory protein precursor; *Haemonchus contortus* | 16 | 782.7 | | 10.6 |
|  |  | gi\|301015486 | Enolase; *Haemonchus contortus* | | 4 | | 191.6 | 16.1 | PPC00777_1 | CE03684 WBGene00011884 locus:enol-1 enolase | 3 | 152.0 | | 2.2 |
| Hc8 | 40kDa | gi\|86161652 | Protein disulfide isomerase; *Teladorsagia circumcincta* | | 9 | | 644.3 | 23.3 | HBC00270_1 | Protein disulphide isomerase; *Ancylostoma caninum* | 9 | 507.7 | | 4.4 |
|  |  | gi\|46249431 | Protein disulfide isomerase; *Ancylostoma caninum* | | 8 | | 474.6 | 14.2 | OOC00150_1 | Protein disulphide isomerase; *Teladorsagia circumcincta* | 8 | 521.8 | | 7.8 |
|  |  | gi\|308511555 | CRE-PDI-2 protein; *Caenorhabditis remanei* | | 6 | | 371.9 | 11 | HCC00273_1 | Protein disulphide isomerase; *Teladorsagia circumcincta* | 7 | 487.8 | | 13.9 |
|  |  | gi\|45752338 | Aspartyl protease precursor; *Haemonchus contortus* | | 4 | | 208.3 | 9.1 | HCC01001_1 | Cathepsin D-like aspartic protease; *Ancylostoma ceylanicum* | 4 | 275.6 | | 8.2 |
|  | | | | | | | | | HCC02381_1 | CE03912 WBGene00003053 locus:lmp-1 | 3 | 152.6 | | 5.3 |
|  | | | | | | | | | HCC00429_1 | Aspartyl protease precursor; *Haemonchus contortus* | 2 | 128.7 | | 3.0 |
| Hc9 | 35kDa | gi\|341864437 | Galectin 5; *Angiostrongylus cantonensis* | | 4 | | 192.9 | 13.6 | NBC01256_1 | Galectin protein 5; *Caenorhabditis elegans* | 5 | 210.9 | | 5.4 |
|  | | | | | | | | | TDC02877_1 | CE29634 WBGene00002268 locus:lec-5 | 5 | 196.0 | | 5.1 |
| Hc10 | 22kDa | gi\|56182492 | 24 kDa excretory/secretory protein; *Haemonchus contortus* | | 6 | | 346.7 | 21.3 | HCC00308_1 | 24 kDa excretory/secretory protein; *Haemonchus contortus* | 16 | 835.9 | | 11.1 |
|  |  | gi\|334306137 | P24; *Haemonchus contortus* | | 3 | | 170.2 | 27.0 | HCC00071_1 | 24 kDa excretory/secretory protein; *Haemonchus contortus* | 12 | 624.0 | | 12.2 |
|  | | | | | | | | | HCC00248_3 | Venom-allergen-like protein 1, isoform a; *Caenorhabditis elegans* | 5 | 309.0 | | 5.3 |
|  | | | | | | | | | HCC13141_1 | 24 kDa excretory/secretory protein; *Haemonchus contortus* | 4 | 172.3 | | 8.4 |
|  | | | | | | | | | HCC00583_1 | no annotation | 3 | 167.3 | | 6.5 |
|  | | | | | | | | | HCC03129_1 | CE41075 WBGene00022176 | 3 | 120.4 | | 3.0 |
| Hc11 | 15kDa | No significant hits | | | | | | | HCC02182_2 | no annotation | 6 | 356.5 | | 13.2 |
|  | | | | | | | | | HCC02182_1 | no annotation | 5 | 326.8 | | 11.5 |
|  | | | | | | | | | HCC00372_2 | Putative uncharacterized protein; *Caenorhabditis briggsae* | 4 | 193.5 | | 8.6 |
|  | | | | | | | | | HCC07574_1 | CE01221 WBGene00017128 | 3 | 153.6 | | 12.2 |

*H. contortus* protein extract run on a 4-12% Bis-Tris gel and bands individually analysed by LC-ESI-MS/MS and MASCOT searches against the NCBInr and NEMBASE4 databases. Only significant hits which were not mammalian, trypsin or keratin are shown. Table showing accession numbers, number of peptide matches, MOWSE scores and sequence identities of each protein match.
