# Supplementary material for: Proteomic Analysis of Mecistocirrus digitatus and Haemonchus contortus Intestinal Protein Extracts and Subsequent Efficacy Testing in a Vaccine Trial
Source: PLoS Negl Trop Dis. 2014 Jun 5;8(6):e2909. doi: 10.1371/journal.pntd.0002909 (PMC4046941; doi:10.1371/journal.pntd.0002909)
Supplement: Table S2 — Putative identities of individual bands from the M. digitatus protein extract. M. digitatus protein extract run on a 4–12% Bis-Tris gel and bands individually analysed by LC-ESI-MS/MS and MASCOT searches against the NCBInr and NEMBASE4 databases. Only significant hits which were not mammalian, trypsin or keratin are shown. Table showing accession numbers, number of peptide matches, MOWSE scores and sequence identities of each protein match. (DOCX) [file pntd.0002909.s002.docx]

**Table S2. Putative identities of individual bands from the *M. digitatus* protein extract**

| **Band** | **Approx band size** | **NCBI Results** | | | | | **NEMBASE Results** | | | | |
| --- | --- | --- | --- | --- | --- | --- | --- | --- | --- | --- | --- |
|  |  | **Accession number** | **Putative Identity** | **Peptides** | **MOWSE score** | **Sequence coverage (%)** | **Accession number** | **Putative Identity** | **Peptides** | **MOWSE score** | **Sequence coverage (%)** |
| Md1 | >250kDa | No significant hits | | | | | No significant hits | | | | |
| Md2 | >250kDa | No significant hits | | | | | No significant hits | | | | |
| Md3 | 220kDa | gi\|6012985 | Microsomal aminopeptidase; *Haemonchus contortus* | 8 | 467.9 | 9.9 | HCC00035_1 | Microsomal aminopeptidase; *Haemonchus contortus* | 8 | 467.9 | 2.7 |
|  | | | | | | | HCC00714_1 | L3B25; *Teladorsagia circumcincta* | 3 | 163.3 | 3.1 |
| Md4 | 185kDa | gi\|3415005 | Putative zinc metallopeptidase; *Haemonchus contortus* | 3 | 140.1 | 4.7 | HCC00065_1 | Putative zinc metallopeptidase precursor; *Haemonchus contortus* | 4 | 201.3 | 0.8 |
|  | | | | | | | HCC00714_1 | L3B25; *Teladorsagia circumcincta* | 3 | 186.9 | 4.3 |
|  | | | | | | | CJC03696_1 | Putative uncharacterized protein; *Caenorhabditis briggsae* | 3 | 99.3 | 2.3 |
|  | | | | | | | HCC11346_1 | Putative zinc metallopeptidase; *Haemonchus contortus* | 2 | 91.1 | 5.0 |
| Md5 | 115kDa | gi\|6012985 | Microsomal aminopeptidase; *Haemonchus contortus* | 22 | 1422.7 | 23.9 | HCC00035_1 | Microsomal aminopeptidase; *Haemonchus contortus* | 22 | 1447.6 | 6.1 |
|  |  | gi\|2499899 | Aminopeptidase N | 16 | 1077.2 | 21.3 | HCC00029_1 | Aminopeptidase N; *Haemonchus contortus* | 17 | 1128.2 | 6.4 |
|  |  | gi\|218139543 | Microsomal aminopeptidase H11; *Haemonchus contortus* | 15 | 991.3 | 19.9 | HCC01491_1 | Membrane aminopeptidase H11-4; *Haemonchus contortus* | 10 | 526.4 | 3.1 |
|  |  | gi\|29825703 | Hidden antigen h11; *Haemonchus contortus* | 11 | 800.9 | 18.8 | HCC00036_1 | Microsomal aminopeptidase; *Haemonchus contortus* | 7 | 440.7 | 3.3 |
|  |  | gi\|14140052 | Membrane aminopeptidase H11-4, isoform 4; *Haemonchus contortus* | 9 | 525.6 | 10.6 | HCC00714_1 | L3B25; *Teladorsagia circumcincta* | 3 | 132.7 | 4.0 |
|  |  | gi\|6012987 | Microsomal aminopeptidase; *Haemonchus contortus* | 7 | 476.3 | 10.4 |  | | | | |
| Md6 | 95kDa | gi\|6012985 | Microsomal aminopeptidase; *Haemonchus contortus* | 7 | 478.1 | 12.8 | HCC00035_1 | Microsomal aminopeptidase; *Haemonchus contortus* | 8 | 498.0 | 3.2 |
|  | | | | | | | HCC00714_1 | L3B25; *Teladorsagia circumcincta* | 4 | 241.6 | 5.2 |
| Md7 | 65kDa | gi\|253721985 | Glutamate dehydrogenase; *Haemonchus contortus* | 7 | 349.1 | 14.6 | HCC00006_2 | Putative glutamate dehydrogenase; *Haemonchus contortus* | 7 | 349.1 | 3.5 |
|  |  | gi\|1335976 | Apical gut membrane polyprotein; *Haemonchus contortus* | 4 | 290.6 | 7.1 | HCC00001_2 | Apical gut membrane polyprotein; *Haemonchus contortus* | 5 | 339.9 | 2.0 |
|  |  | gi\|86161652 | Protein disulphide isomerase; *Teladorsagia circumcincta* | 3 | 213.8 | 9.3 | DVC00633_1 | Putative uncharacterized protein; *Dictyocaulus viviparus* | 4 | 253.0 | 2.6 |
|  | | | | | | | HCC00714_1 | L3B25; *Teladorsagia circumcincta* | 3 | 178.9 | 3.1 |
| Md8 | 60kDa | gi\|1335976 | Apical gut membrane polyprotein; *Haemonchus contortus* | 10 | 697.9 | 16.5 | HCC00001_2 | Apical gut membrane polyprotein; *Haemonchus contortus* | 12 | 797.3 | 5.6 |
| Md9 | 45kDa | gi\|219753666 | P100GA2 protein; *Haemonchus contortus* | 5 | 241.3 | 5.0 | No significant hits | | | | |
| Md10 | 40kDa | gi\|229552 | Albumin | 3 | 179.7 | 7.1 | No significant hits | | | | |
|  |  | gi\|17549909 | Aspartyl Protease family member (asp-4); *Caenorhabditis elegans* | 2 | 117.4 | 6.5 |  | | | | |
| Md11 | 36kDa | No significant hits | | | | | HCC00714_1 | L3B25; *Teladorsagia circumcincta* | 13 | 118.7 | 4.0 |
| MD12 | 22kDa | No significant hits | | | | | HCC00714_1 | L3B25; *Teladorsagia circumcincta* | 4 | 260.4 | 5.2 |
| Md13 | 15kDa | No significant hits | | | | | No significant hits | | | | |

*M. digitatus* protein extract run on a 4-12% Bis-Tris gel and bands individually analysed by LC-ESI-MS/MS and MASCOT searches against the NCBInr and NEMBASE4 databases. Only significant hits which were not mammalian, trypsin or keratin are shown. Table showing accession numbers, number of peptide matches, MOWSE scores and sequence identities of each protein match.
